# Individual differences in attributional style but not in interoceptive sensitivity, predict subjective estimates of action intention

**DOI:** 10.3389/fnhum.2014.00638

**Published:** 2014-08-19

**Authors:** Tegan Penton, Guillaume L. Thierry, Nick J. Davis

**Affiliations:** ^1^Department of Psychology, Goldsmiths, University of LondonLondon, UK; ^2^School of Psychology, Bangor UniversityBangor, UK; ^3^Department of Psychology, Swansea UniversitySwansea, UK

**Keywords:** W-judgment, libet, interoception, locus of control, agency

## Abstract

The debate on the existence of free will is on-going. Seminal findings by Libet et al. (1983) demonstrate that subjective awareness of a voluntary urge to act (the W-judgment) occurs before action execution. Libet’s paradigm requires participants to perform voluntary actions while watching a clock hand rotate. On response trials, participants make a retrospective judgment related to awareness of their urge to act. This research investigates the relationship between individual differences in performance on the Libet task and self-awareness. We examined the relationship between W-judgment, attributional style (AS; a measure of perceived control) and interoceptive sensitivity (IS; awareness of stimuli originating from one’s body; e.g., heartbeats). Thirty participants completed the AS questionnaire (ASQ), a heartbeat estimation task (IS), and the Libet paradigm. The ASQ score significantly predicted performance on the Libet task, while IS did not – more negative ASQ scores indicated larger latency between W-judgment and action execution. A significant correlation was also observed between ASQ score and IS. This is the first research to report a relationship between W-judgment and AS and should inform the future use of electroencephalography (EEG) to investigate the relationship between AS, W-judgment and RP onset. Our findings raise questions surrounding the importance of one’s perceived control in determining the point of conscious intention to act. Furthermore, we demonstrate possible negative implications associated with a longer period between conscious awareness and action execution.

## INTRODUCTION

The concept of free will has long been a controversial topic in both philosophical and scientific domains (Sinnott-Armstrong and Nadel, 2011). Here free will, or volitional action, is defined as conscious awareness of the intention to act. The traditional concept of free will (control of one’s actions) has been challenged by the research of Libet et al. (1983); whose results show onset of neural activity associated with an action before an individual becomes aware of their intention to act. In their seminal experiment, Libet et al. (1983) used EEG to record the readiness potentials (RP) of six participants while they completed a computer task. During the task, participants were asked to watch a clock hand rotate around a clock and to press a button only if they felt the urge to act (to emphasize voluntary action). If a response was made during a given trial, the participant was asked to indicate the position of the clock hand when they first became aware of the urge to move (known as the W-judgment). The RP (or Bereitschaftspotential) is characterized by a slow negative shift in potential related to the motor and pre-motor area (Luder Deecke and Kornhuber, 1969) and is often seen before voluntary movements (for example, Waszak et al., 2005; for alternative explanations see Schurger et al., 2012). Libet et al. (1983) showed that, on average, an RP was seen 550 ms before action initiation while W-judgments were seen 206 ms before action initiation (-206 ms). Therefore, Libet et al. (1983) suggested that action intention is not entirely “free” and that conscious awareness may occur as more of a justification of a predetermined action.

Libet (1999) later argued that these findings do not necessarily negate the concept of volition, rather the phenomenon may exist in the period between awareness of the urge to act and action execution. Specifically, Libet (1999) suggested that 200 ms latency between awareness and action execution could allow for conscious inhibition of that action if required. This latency is known as the “veto” period and is used to provide a more observable notion of volition (Haggard and Libet, 2001; Mele, 2008). While the Libet paradigm has been subject to criticism (see Haggard et al., 2002), research accounting for issues related to task constraints (Matsuhashi and Hallett, 2008; RP’s 1.42 s prior to action onset) and subjective report (Fried et al., 2011; activity seen 700 ms prior to action onset in single-cell recordings) still replicate the basic findings of Libet’s work. However, the precise timing of associated neural activation is disputed (for more replications see, Lau et al., 2004; Soon et al., 2008).

In spite of the wealth of research into the Libet paradigm, the influence of individual differences in response patterns on the Libet task is relatively unknown. Libet et al. (1983) did take individual differences in response patterns into account (by creating a discrepancy score between a participants average W-judgment and the average time of perceived external touch, determined by another task) in the hope of providing a more reliable estimate of awareness of intention to act, but did not consider other inter-individual differences (e.g., personality). Haggard and Eimer (1999) also addressed variance in W-judgments by investigating variance within a participant’s W-judgments and the covariance of associated brain activity (namely, the RP and lateralised RP; a potential calculated by investigating the relative shift in activity between the contralateral and ipsilateral hemisphere to the hand performing the action). They suggest that LRP onset covaries with time of W-judgments in that early W-judgments correlate with early LRP onset and late W-judgments correlate with late LRP onset. In this way, it is clear that research into volition is aware of potential individual differences in the W-judgment. The current research aims to investigate the relationship between aspects of self-awareness (IS, one’s awareness of one’s internal stimuli), perceived control (AS; the style one uses to explain life events) and one’s awareness of one’s intention to act. To our knowledge this is the first research investigating personality and perceptual correlates of W-judgments on the Libet task.

Attributional style (AS) refers to the style an individual uses to explain previous positive and negative life events. Peterson et al. (1982) developed the AS Questionnaire (ASQ) to measure perceived control across several modalities. In order to enable a more holistic understanding of an individual’s perception of control to be established, the ASQ attempts to define the style that individuals adopt to explain life events across three areas; (1) Internality (whether the individual feels the cause of the event is due to themselves or an external factor), (2) Stability (whether the individual feels this cause is stable over time), and (3) Globality (whether the individual feels the cause will be present across multiple life domains). Those who view the cause of positive life events as internal, stable and global, and the cause of negative life events as external, transient, and specific are said to have a positive or optimistic AS; while those who view the cause of positive life events as external, transient, and specific, and the cause of negative life events as internal, stable and global are thought to have a negative or pessimistic AS. Many benefits of having an optimistic AS have been reported in the literature, such as higher levels of well-being in comparison to those with a negative AS (see Forgeard and Seligman, 2012 for a review). Research into negative AS is more extensive (Seligman et al., 1999; Seligman, 2002) with many reporting a relationship between depression (e.g., Peterson et al., 1982; Stange et al., 2013) and anxiety (e.g., Luten et al., 1997; Mark and Smith, 2012) and negative AS scores. Furthermore research has also shown negative feelings and emotions to correlate with other measures. For example, Critchley et al. (2004) show a positive relationship between “negative emotional experience” and IS.

Interoception refers to one’s awareness of one’s internal stimuli (e.g., an individual’s ability to estimate their own heartbeats over a given time period, Craig, 2002). The somatic-marker hypothesis proposed by Damasio et al. (1996) suggests that emotional and physiological changes elicited by exposure to certain situations or stimuli are bound together. Therefore, when encountering a new stimulus that elicits the same physical arousal/emotion, the individual will evaluate the potential reward or punishment based on prior experience. Werner et al. (2013) supports this theory by showing that increased interoceptive awareness relates to increased processing of somatic markers during a decision making task. Craig (2004) suggests this integration of interoceptive and emotion information occurs within a neural network converging in the insular cortex. Furthermore, he later suggests that integration of this information occurs at each moment in time to create a global, time-locked, sense of self-awareness (Craig, 2010). Relating this to the current research, work by Berlucchi and Aglioti (2010) demonstrate that similar cortical regions, primarily the Insula, are associated with both interoception and agency (a sense of control over one’s actions). In this context, one may expect a relationship between performances on the Libet task, AS and IS in the current study.

There is evidence to suggest that perceived control and belief in free will are related, with Baumeister and Brewer (2012) demonstrating a positive correlation between internal Locus of Control (attribution of the cause of life events to the self; LOC; Rotter, 1966) and belief in free will. Furthermore, Stroessner and Green (1990) demonstrate a positive correlation between beliefs in determinism and external LOC (attribution of the cause of life events to external factors). Supporting this, Paulhus and Carey (2011) demonstrate a positive correlation between belief in free will and AS (one’s style of explaining life events; a measure of perceived control). As well as this, Orellana-Damacela et al. (2000) suggest that, when more self-aware, one is more likely to consult one’s own standards and beliefs during decision-making. It is proposed that this act can be beneficial or detrimental to the individual in question based on their ability to meet their own expectations. This suggests that individual differences in levels of self-awareness can have varying effects on cognition based on top-down factors such as perceived control and decision making. However, little is known about the relationship between one’s conscious awareness of intention to act and one’s perceived control over life events. Rigoni et al. (2011) attempt to address this issue by investigating the neural correlates associated with manipulating belief in free will. Participants who read a passage of text negating the concept of free will showed decreased RP amplitude, but not W-judgment latency, during the Libet task in comparison to those who read a neutral passage of text. Rigoni et al.’s (2011) work demonstrates the relationship between neural activity associated with action execution and higher level beliefs while demonstrating the malleability of both. However, it is still unclear to what extent pre-existing perceptions of control and awareness of conscious intention to act are related. Therefore, the current research aims to investigate how individual differences in perceived control and self-awareness correlate with one another and with awareness of intention to act.

## MATERIALS AND METHODS

### ETHICAL APPROVAL

Prior to data collection, ethical approval was granted by Bangor University’s Ethics Board. All participants were recruited via the universities recruitment site and were offered printer credits or course credits as compensation for taking part. Written consent was obtained from all participants before beginning the experiment.

### TRIALS AND PROCEDURE

A repeated measures design was used to allow for correlational data analysis and to reduce inter-subject variance. Analysis consisted of a multiple regression to assess whether AS and IS predicted performance on the Libet task. A separate correlation was run using Interoceptive sensitivity scores and AS scores. All tasks (clock, questionnaire, and heart-rate) were counterbalanced across participants.

#### Clock task

The stimuli used were similar to that of Libet et al. (1983), consisting of a black clock hand rotating around a clock-like object on a white background (stimuli remained on screen during inter-trial intervals). The clock hand disappeared during the judgment part of the task (see Procedure). During each trial the clock hand rotated around the clock 3 times (2 s per rotation, 6 s in total). The hand completed three full rotations for every trial (including response trials) to prevent the stop position of the hand from influencing the W-judgment. Participants were instructed to allow one full rotation of the clock hand around the clock and to click the mouse at any point during the final two rotations if they felt the urge to do so. On response trials, following three rotations of the clock hand, the clock hand disappeared and a question mark appeared in the middle of the screen. The participant was instructed to use the mouse to make a retrospective judgment of when they first became aware of the urge to act. “Using the mouse, please mark the point on the clock that the clock hand was at when you first became aware of the urge to act.” The next trial began once a mouse click was detected. Trials where no response occurred were excluded from the final analysis. There were 60 trials during the task but, due to the voluntary nature, there was variation in the number of trials included for each individual.

#### Interoceptive sensitivity task

Participants’ heart beat estimates were recorded as well as actual heart beats using an electrocardiogram (electrodes were attached to both wrists and one ankle of the participant). The task consisted of six blocks of varying length (35 s, 45 s, 100 s, repeated) in a randomized order across participants to allow for reliable and varied estimates between participants. Intervals between blocks also varied in length (75 s, 65 s, 55 s and immediate start) – these were also randomized across participants. Participants were instructed to count their heart beats to the best of their ability without taking their pulse. Participants were instructed to close their eyes throughout the experiment and to count their heartbeats to the best of their ability without taking their pulse. Upon hearing a single tone, they were to start counting, upon hearing two short tones; they were required to verbally report the number of heartbeats they had counted.

#### ASQ

Participants were required to answer the 12 items on the ASQ. Each item consisted of a scenario (for example, “You meet a friend who acts hostilely toward you”) followed by four questions (one qualitative – “Write down one major cause for this event”) – the questions were the same for all items. The participant was required to give an example of one major cause for the scenario and to rate this cause across three, 7-point, likert scales to assess internality (“Is this cause due to something about you or to something about other people or circumstances?”), stability (“In the future, will this cause again be present?”) and globality, respectively (“Is the cause unique to this situation or does it also influence other areas of your life?”).

### DATA ANALYSIS

#### Clock task

Only data from response trials was included in the analysis. If number of response trials were more than 2 SD away from the mean, that participant’s data was excluded from analysis. The angle of the clock-hand on the clock when the participant made a button press was recorded as well as the angle the mouse was at during the judgment phase of the task. Both angles were converted into time by dividing the angle score by π. To obtain the difference scores, the time of action was taken from the W-judgment time to produce a negative number. Therefore, the closer the difference score was to 0, the smaller the distance between action execution and W-judgment.

#### Interoceptive sensitivity task

The following formula was used to calculate an average accuracy score (scores were summed for all six trials prior to entry into the formula):

∑1−[|Recorded⁢ Heartbeats−Counted⁢ Heartbeats|Recorded⁢ Heartbeats]

This was then multiplied by 100 to give a percentage accuracy score. Participants who provided more accurate estimates had a higher accuracy score thought to be indicative of better interoceptive sensitivity (Schandry, 1981).

#### ASQ

It is worth noting that the questionnaire’s subscale reliability is low (internality, *r* = 0.54; stability, *r* = 0.65, globality; *r* = 0.59; Peterson et al., 1982), however, when compounding the scales together, the reliability is vastly improved (positive AS, *r* = 0.75, negative AS, *r* = 0.72). As we were concerned with a holistic representation of perceived control, we analyzed response on the questionnaire by taking an average across all the scales for positive and negative questions, respectively. To obtain an overall AS score for each participant, scores from all three subscales for each of the six questions with a positive valence were summed and divided by 18, the same was done for the six questions with a negative valence. The negative composite score was then taken from the positive composite score to obtain an overall composite score of attributional style. Higher scores were indicative of a more positive AS (more likely to attribute positive events to internal, stable, global attributes, and negative events to external, transient, specific attributes).

## RESULTS

Three participants were removed due to incomplete data on the heartbeat task (electrode recordings were too noisy) and two participants were removed due to outlier data (one for only completing six trials on the Libet task, and 1 because of an average W-judgment further than 2 SD from the group mean). Due to the voluntary nature of the Libet task, the number of trials completed varied between participants (responses *M* = 45.84, SD = 12.91). Data for 25 participants (13 female, Mean age = 23.6, range = 20–39) was included in the analysis. Descriptive statistics from the Libet task represent the latency between W-judgment and action execution in milliseconds (this is a negative number as awareness occurred before action onset in all participants), while percentage scores were used for data from the heartbeat task and composite scores were used to represent performance on the attributional style questionnaire (see **Table 1**).

**Table 1:** Descriptive statistics for W-judgment, AS score and heartbeat accuracy.

	W-judgment (ms)	ASQ (7-point Likert)	Heartbeat accuracy (%)
Mean (SD)	-253ms (198 ms)	0.29 (1.06)	65.93 (15.84)
Min/Max	-640/-30	-2.28/1.78	36.41/96.96

*Values in parentheses indicate standard deviation.*

### PREDICTION OF MEAN W-JUDGMENT FROM AS AND IS SCORES

**FIGURE 1:**
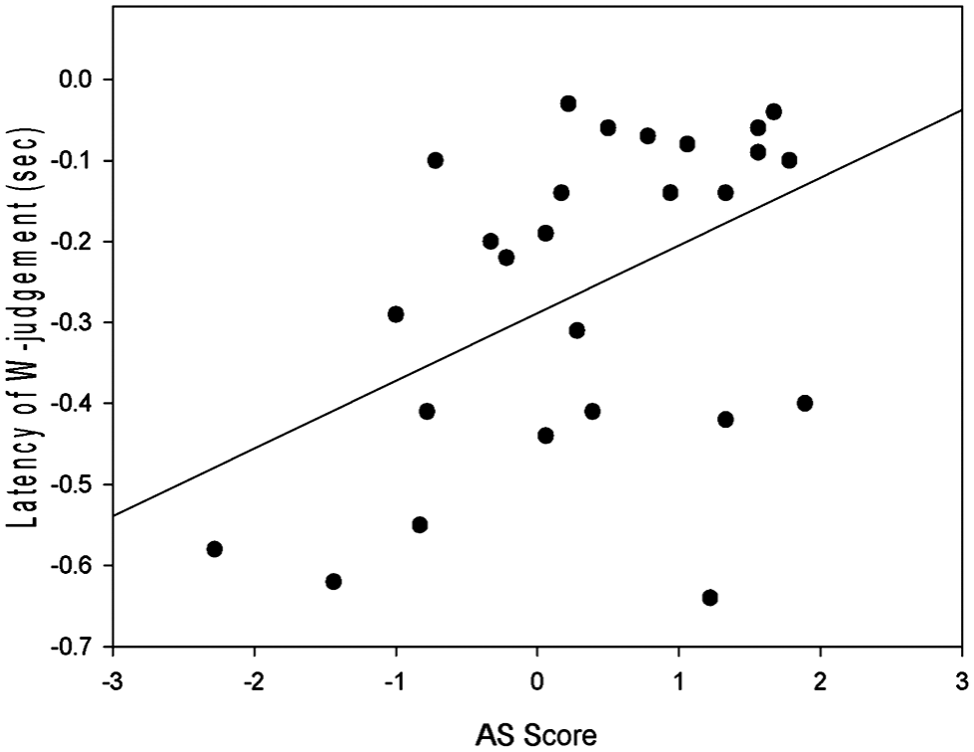
**Prediction of Mean W-judgment scores from attributional style scores (AS Score), with linear regression (*R^2^* = 0.32, *p* = 0.015)**.

A multiple regression was conducted to establish the relationship between performance on the Libet task, heartbeat accuracy and AS scores. The “Mean W-judgment” variable was used as the outcome variable with the “Attributional Style” and “Heartbeat Accuracy” variables acting as predictors. Predictor variables were entered using the forced entry method due to the exploratory nature of the research. Diagnostic tests did not reveal any violations of the test statistics. Multi-collinearity between predictor variables was not observed during diagnostic tests in the multiple regression (*VIF* = 1.22, *Tolerance* = 0.82) and normality was assumed. The regression model was found to be significant (*R*^2^ = 0.32, *F*(2,22) = 5.08, *p* = 0.015) suggesting that the two predictor variables (“Heartbeat Accuracy” and “Attributional Style”) explained 31.6% of the variance (see **Table 2**). ASQ score was a significant predictor of mean W-judgment but heartbeat accuracy score was not (see **Figure 1**).

**Table 2:** The unstandardised (u) and standardized (s) beta coefficients as predictors of W-judgment.

Variables	B (u)	β (s)	*p*
ASQ (SE)	0.115 (0.036)	0.617	0.004
Heartbeat accuracy (SE)	0.002 (0.002)	0.198	0.321

*Values in parentheses represent the standard error. R^2^ = 0.32 (p = 0.015).*

### RELATIONSHIP BETWEEN AS AND IS

**FIGURE 2:**
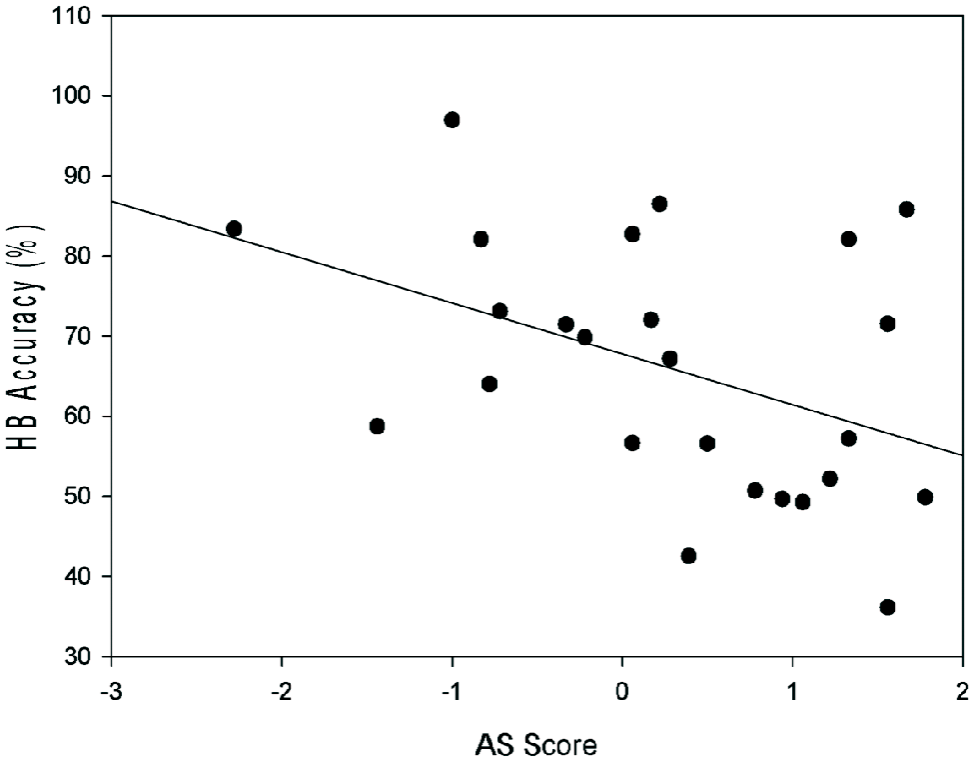
**The relationship between AS Score and heartbeat accuracy score (%; HB Accuracy), *r* = -0.43, *p* = 0.034**.

A separate correlation was run to investigate the relationship between “Attributional Style” and “Heartbeat Accuracy”. A medium negative correlation was observed at a 2-tailed significance level, *r*(23) = -0.43, *p* = 0.034 (see **Figure 2**).

## DISCUSSION

The results indicate that, while performance on the ASQ can predict performance on the Libet task (consistent with our predictions), IS was not a significant predictor of Libet performance, contrary to our predictions. Specifically, more negative AS scores correlate with more negative W-judgments (further away from action onset). A significant relationship was also observed between AS score and IS.

Firstly, it is important to note that this research serves as a replication of Libet et al. (1983) original findings in that the mean W-judgment across the entire sample (*M* = -253 ms) was similar to that of Libet’s sample (*M* = -206 ms). This is also consistent with other replications of the Libet experiment for example, Lau et al. (2004) reported a *M* = -228 ms while investigating fMRI correlates of voluntary action and Rigoni et al. (2011) also approximately replicate Libet’s findings while demonstrating that reducing belief in free will correlates with significant reduction in early RP amplitude, but not with a change in W-judgment (Reduced belief group *M* = -242, Control group *M* = -223). As our data is consistent with the literature, it is possible that individual differences in AS may have had undetected effects on previous findings in the same way as the current research. The large variance of W-judgment values in the literature may be indicative of these individual differences (i.e., Libet et al., 1983). Furthermore, given the direction of the previous literature (for example, Libet et al., 1983; Matsuhashi and Hallett, 2008), it is safe to presume that an overall average W-judgment of -253 ms will follow onset of the RP by several hundred milliseconds.

More negative mean scores are indicative of a larger discrepancy between W-judgment and action execution. This would suggest that those with a more negative AS may be aware of the intention to act sooner than those with a positive AS. It may also be that W-judgment accuracy is affected by these top-down personality factors. This suggests that, even if criticisms surrounding the paradigm were addressed; such as those related to reliance on recall of the urge to act, (for example, Dennett and Kinsbourne, 1992), personality variants may still affect awareness of the urge to act.

This research raises questions surrounding belief in free will – i.e., that a larger veto period may relate to a pessimistic AS. It may be that individuals with a more negative AS may perceive themselves as having less control (and, therefore, less free will) due to a disassociation between action awareness and action execution. Marcel (2003) argues that ownership of action can be separated into ownership of action execution and ownership of action intention. Therefore, a temporal dissociation between the two may reduce the ownership one feels over action execution. In turn, this may lead to a perceived lack of control as intention in the individual’s schema is not bound to execution.

It is possible that those with a more pessimistic AS may be more uncertain in the choices they make, as is consistent with research into pessimistic AS (e.g., Bunce and Peterson, 1997; Boudreaux and Ozer, 2013), while those with a more positive AS are more likely to claim ownership over the action resulting in a smaller latency between W-judgment and action onset. Therefore, the pattern in the W-judgments may simply reflect level of self-doubt and uncertainty in those with a negative AS. This theory is consistent with research into negative AS and self-doubt (Heppner et al., 1985; Bunce and Peterson, 1997).

It is most likely that the relationship observed between AS and W-judgment is heavily influenced by aspects of internality (i.e., “is the cause of a life event due to the individual or to an external factor?”). This was not assessed specifically because of the desire to investigate the relationship between a more holistic representation of perceived control and awareness of intention to act. Furthermore, the poor subscale reliability of the ASQ meant that this relationship was not explored in an additional analysis. However, future research should also employ the LOC questionnaire to assess whether individuals with larger latency between W-judgment and action execution have a more external LOC independent of valence. Furthermore, research should investigate whether those with a positive AS will experience greater ownership over their actions than those with a negative AS. To our knowledge, this research is the first to consider the possible negative implications of having a longer “veto” period. Traditional literature into volition implicates the veto period in conscious control of action (Libet, 1999; Mele, 2008), however, until now, no research has investigated individual differences in the veto period. If the above theory is true, it may be that a larger veto period (indicative of greater control over one’s actions) correlates with reduced levels of perceived control.

The regression analysis demonstrated that IS did not predict awareness of conscious intention to act. However, the results indicate a medium, negative correlation between IS and AS suggesting that the more negative (or pessimistic) an individual’s AS, the better they are at estimating their own heartbeats. Both AS and IS have been shown to correlate with anxiety (Domschke et al., 2010; Mark and Smith, 2012). Therefore, the effect here may relate to a hyper-awareness seen in those with anxiety disorders. It is also possible that, due to the correlation with depressive symptoms (Seligman, 2002), those with a negative AS have a tendency to self-evaluate and, therefore, are more self-aware. It is important to note that researches into the correlates of IS are inconsistent, so more work is still needed in the area (see der Does et al., 2000).

Future research should focus on furthering understanding of individual differences in performance on the Libet task (and other tasks related to awareness of conscious intention to act), and what these differences relate to. More specifically, a causal relationship between AS and W-judgment should be investigated by attempting to manipulate AS (for example, see Anderson, 1983) score and, in turn, modulate performance on the Libet task. This could establish whether perceived control over positive and negative life events may have a causal impact on awareness of conscious intention to act. Manipulating AS score could also be used to investigate a causal relationship between AS and IS. Further investigation is required to uncover latent variables which may modulate the relationships in question. These findings would be strengthened by using EEG to investigate potential neural correlates, specifically the LRP.

Implications of this research are potentially wide ranging; specifically this research informs literature relating to agency, action ownership and AS. Additionally, this research takes a step toward understanding individual differences in awareness of intention to act. More generally, this research suggests that perceived control and volition are related.

In conclusion, it is clear that a relationship exists between performance on the Libet task and performance on the ASQ. It is possible therefore, that some of the variance in the Libet task results from individual differences in top-down traits such as personality variants. The current research highlights potential confounds in the W-judgment related to fluctuations in AS. Furthermore, this research demonstrates that, those with a more negative AS may have a larger latency between W-judgment and action onset. It is proposed that this relationship may result from a discrepancy between conscious awareness of the intention to move, and the consequence of this (action onset) suggesting, for the first time, potential negative implications of a longer veto period.

## Conflict of Interest Statement

The authors declare that the research was conducted in the absence of any commercial or financial relationships that could be construed as a potential conflict of interest.
